# Decision-making under risk and theory of mind in adolescent offenders in provisional deprivation of liberty

**DOI:** 10.47626/2237-6089-2020-0155

**Published:** 2022-11-09

**Authors:** Rubens José Loureiro, Flavio Takemi Kataoka, Thiago Wendt Viola, Gisele Iesbich Vargas, Breno Sanvicente-Vieira, Rodrigo Grassi-Oliveira, Bruno Kluwe-Schiavon

**Affiliations:** 1 Departamento de Medicina e Enfermagem Escola Superior de Ciências Santa Casa de Misericórdia de Vitória Vitória ES Brazil Departamento de Medicina e Enfermagem , Escola Superior de Ciências da Santa Casa de Misericórdia de Vitória , Vitória , ES , Brazil .; 2 Laboratório de Neurociência Cognitiva do Desenvolvimento Pontifícia Universidade Católica do Rio Grande do Sul Porto Alegre RS Brazil Laboratório de Neurociência Cognitiva do Desenvolvimento , Pontifícia Universidade Católica do Rio Grande do Sul (PUCRS), Porto Alegre , RS , Brazil .; 3 Escola de Ciências da Saúde e da Vida PUCRS Porto Alegre RS Brazil Escola de Ciências da Saúde e da Vida , PUCRS , Porto Alegre , RS , Brazil .; 4 Instituto do Cérebro do Rio Grande do Sul PUCRS Porto Alegre RS Brazil Instituto do Cérebro do Rio Grande do Sul , PUCRS , Porto Alegre , RS , Brazil .; 5 Laboratório de Pesquisa em Diferenças Individuais e Psicopatologia Pontifícia Universidade Católica do Rio de Janeiro Rio de Janeiro RJ Brazil Laboratório de Pesquisa em Diferenças Individuais e Psicopatologia , Pontifícia Universidade Católica do Rio de Janeiro (Puc-Rio), Rio de Janeiro , RJ , Brazil .; 6 Departamento de Psicologia Puc-Rio Rio de Janeiro RJ Brazil Departamento de Psicologia , Puc-Rio , Rio de Janeiro , RJ , Brazil .; 7 Centro de Investigação em Ciências Psicológicas Universidade de Lisboa Portugal Decisão em Contexto, Centro de Investigação em Ciências Psicológicas, Universidade de Lisboa, Alameda da Universidade, Portugal.

**Keywords:** Social cognition, decision-making, risk taking, delinquency, criminality

## Abstract

**Introduction:**

Delinquent behaviors are risky behaviors that increase during puberty and reach their highest peak in late adolescence. It has been proposed that poor decision-making and theory of mind (ToM) are key cognitive processes implicated with delinquency during adolescence, affecting evaluation of risks and impairing appreciation of social norms. Nevertheless, it is not yet clear whether adolescent offenders who are subjected to provisional deprivation of liberty due to conflict with the law (adolescents in conflict with the law [ACL]) might, in fact, present a specific profile with regard to these cognitive processes.

**Objectives:**

To assess deliberative decision-making and ToM among adolescents in conflict with the law and adolescents not in conflict with the law.

**Methods:**

The sample comprised 62 participants: ACL (n = 29) and a control group (CG) (n = 33). ToM was assessed with the Reading the Mind in the Eyes Test (RMET) and decision-making was assessed with the Columbia Card Task (CCT). Substance use, callous-unemotional traits, childhood maltreatment, and intelligence quotient (IQ) were also assessed.

**Results:**

ACL had more ToM errors for negative mental states in comparison to CG, but not for error rates concerning neutral and positive mental states. With regards to decision-making, our results suggest that ACL group members did not vary their behavior based on the available information and that the risk information had an opposite effect on the number of cards chosen (risk-taking behavior) when compared to CG.

**Conclusion:**

These findings have important implications for development of interventions for these adolescents, suggesting that they tend to learn little from negative outcomes and have reduced capacity to process negative emotions.

## Introduction

It has been exhaustively shown that risk-taking behaviors such as use of licit and illicit substances, unsafe sexual practices, aggressive behaviors, and risky recreational sports increase during puberty, reaching their highest peak in adolescence, and decrease along adulthood. ^1^ Nevertheless, for many individuals, these behaviors might not fade during development, but turn into recurrent delinquency that leads them into problems with the law. The number of child delinquents entering the juvenile justice system is a serious worldwide problem, raising fiery political debates and challenging researchers in the social sciences, law, and health. Recent data showed that there were more than 22,000 adolescents in detention in Brazil, with approximately 18,300 already sentenced, corresponding to an incidence of 8.8/100,000 per inhabitants. ^2^ Several social, economic, cultural and biological factors have been identified as risk factors for many emotional and behavioral problems during the transition from adolescence into adulthood, ^3^ increasing adolescents’ vulnerability to delinquent behavior and infraction. Therefore, beyond social vulnerability, which covers, not exclusively, marginalization, structural disadvantage, economic inequality, and childhood maltreatment, ^4^ cognitive developmental factors related to decision-making and inadequate reason about our own thoughts, feelings, and intentions have been widely related to criminal behavior among teenagers. ^5^ In this regard, proponents of dual-processing models of information processing have paid particular attention to a recently proposed framework of criminal decision-making, explaining, at least in part, how decisions could be triggered by an imbalance between an emotional “hot” neural system and a deliberative “cool” cognitive neural system. ^5^

In a broad sense, all decisions we make result, at some level, from the individual’s capacity to properly identify possible alternatives and to evaluate them based on environmental contingencies, finally choosing the one with the highest utility. ^6^ Value-based normative decision-making approaches suggest that individuals should choose the alternative in accordance with their beliefs about the expected value of that alternative, in which an optimal strategy would take into account gains, losses, and probabilities (i.e., risk). ^7^ Thus, deliberative decision-making under risk, in which all the necessary information is available, refers to those situations in which it is possible to evaluate losses and gains for each alternative identified, as well as the risks involved without social contingencies. Deliberative decision-making is highly dependent on individual factors such as sex, ^8^ neuropsychiatric conditions, developmental stages ^1 , 9^ and early life experiences, such as parenting and stress. ^10^ For example, it has been suggested that adults with high levels of impulsivity and executive function impairments present increased risky behavior in unfavorable deliberative decision-making scenarios. ^11^

Additionally, aspects related to social cognition (e.g., morality, empathy, and how people care and handle social environmental stimuli) have always been guiding factors in social balance and in the construction of legal norms and may be compromised in adolescents in conflict with the law (ACL). A major process of social cognition is theory of mind (ToM), which refers to the ability to infer and understand the emotions, intentions, thoughts, and actions of others. ^12 , 13^ Hence, similar to what is proposed by value-based decision approaches, in which environmental contingencies such as the magnitude of losses and gains contingent on each of the alternatives identified might directly influence one’s decision, ToM is highly dependent on the dynamic perception of facial cues during group interaction that might guide social value orientation and behaviors. In this regard, social cognition is also an important correlate of behavioral impulsivity and violence among adults and neuropsychiatric patients. ^14 , 15^ Although there is evidence that adults with criminal records with and without associated mental illnesses might perform worse on ToM tasks, ^16 , 17^ some studies are inconclusive ^14 , 18^ and few studies have explored this ability among adolescents accused of committing a crime.

Altogether, considering that (i) social vulnerability related to adverse developmental experiences is associated with crime initiation and maintenance, with a peak rate of criminal justice reports among those in the late years of adolescence ^19^ ; that (ii) risky behaviors might reflect, at least in part, impairments to the capacity to properly evaluate losses, gains, and risks for each alternative identified; that (iii) ToM impairments might be associated with behavioral impulsivity, violence, and involvement in criminal activity; and (iv) that few studies have focused on deliberative decision-making under risk and social cognitive features in adolescent offenders; the primary aim of this study was to assess deliberative decision-making and ToM among adolescent offenders who are subject to provisional deprivation of liberty (temporary detention) due to conflict with the law and a control group (CG) of adolescents who are not. Moreover, although value-based decision-making and social cognition are distinct cognitive processes and ontogenetic behaviors, their relationship has not yet been fully explored.

## Method

### Participants and procedure

A total sample of 100 participants (58 ACL, and 42 CG) was assessed initially. However, after applying exclusion criteria, a final sample of 62 participants (29 ACL and 33 CG) was actually analyzed. The ACL group consisted of adolescent offenders who were taken into socio-educational measures at a provisional social rehabilitation center run by the Instituto de Atendimento Socioeducativo do Espírito Santo (IASES), in southeastern Brazil, in 2019. Provisional deprivation of liberty lasts a maximum of 45 days while the adolescent awaits a judicial decision. During this period, the adolescent is enrolled in school, attends classes, and is entitled to sports and leisure programming. The CG members were recruited at public schools.

The inclusion criteria for all groups were: (i) to be able to read, understand and provided written informed assent (for the ACL, the institution granted consent, while consent for adolescents under 18 in the CG was given by their parents); (ii) aged between 16 and 19 years; and (iii) male sex. Exclusion criteria were: (i) self-report of presence of any neurological disorder or brain injury; (ii) intake of medication with potential action on the autonomic or central nervous systems during the last seven days; (iii) self-report of current diagnoses of infectious diseases or severe somatic disorder; (iv) severe cognitive deficit (intelligence quotient [IQ] < 65 based on the Wechsler Abbreviated Scale of Intelligence [WASI] and/or a total score < 18 on the Mini-Mental State Examination [MMSE]); (vi) less than 5 years of education; (vii) self-report history of psychosis or chronic psychiatric disorders; or (viii) acute intoxication by licit or illicit drugs or withdrawal symptoms.

All adolescents provided written informed assent in accordance with the Declaration of Helsinki, and written-informed consent for the CG was obtained from their parents or legal guardian. ACL only provided informed assent, since they were in a provisional social rehabilitation center. Nevertheless, the institution gave consent for the ACL group. The study was approved by the Ethics Committee at the Escola Superior de Ciências da Santa Casa de Misericórdia de Vitória, a higher education institution located in Espírito Santo, Brazil, and registered with the National Research Ethics Council (2.917.120). Moreover, the study was also approved by the IASES Information Center, which regulates research with adolescents who are under socio-educational measures at provisional social rehabilitation centers, in accordance with the Children and Adolescents’ Statute (Estatuto da Criança e do Adolescente) and Brazilian Federal Law 12,594/2012. ^20 , 21^

### Clinical and cognitive assessment

IQ was estimated using both the Vocabulary and the Matrix Reasoning tests from the WASI, ^22^ while general cognitive functioning (orientation, attention, memory, language, and visual-spatial skills) was assessed with the MMSE. ^22 , 23^ Substance consumption was assessed with the CRAFFT Screening Test. ^24 , 25^ The CRAFFT screens for alcohol, cannabis, and other drugs and its questions were designed to be developmentally appropriate for adolescents. In addition to assessing the prevalence of substance use (Criteria A score), the questionnaire also allowed us to assess whether the participants already had alcohol or other drug-related problems (Criteria B score). Moreover, a score of 2 has been identified as the optimal threshold for identifying a possible risk for substance use disorders. ^26^ The Juvenile Victimization Questionnaire second revision (JVQ-R2) was used to assess crime, child maltreatment, and other kinds of victimization experiences during childhood. ^27^ The JVQ-R2 contains screening questions about 34 offenses against youth that cover five general areas of concern, i.e., conventional crime, child maltreatment, peer and sibling victimization, sexual victimization, and witnessing and indirect victimization. Additionally, the Inventory of Callous-Unemotional Traits (ICU) was used to investigate possible antisocial and/or aggressive youth. ^28^ This 24-item questionnaire has three subscales: callousness, uncaring, and unemotional.

### Decision-making: Columbia Card Task (CCT)

Due to our focus on understanding how participants deliberatively evaluate environmental information such as gains, losses, and risk to make decisions, all participants performed the no-feedback condition of the CCT. ^1 , 11 , 29 - 31^ In this task, participants are shown a deck with 32 cards placed facedown and three explicit pieces of information: how many losing cards are hidden in the deck (i.e., risk 1 or 3), the amount associated with each losing card (i.e., a loss of −250 or −750 points), and the amount associated with each winning card (i.e., a gain of 10 or 30 points). In each round, participants choose how many cards the computer will randomly select and turn over, knowing that the round will end immediately if the computer selects one of the losing cards. The different combinations of gain, loss, and risk culminate in eight possible decision scenarios that can be sorted from the most favorable (i.e., risk = 1, loss = -250, gain = 30) to the least favorable (i.e., risk = 3, loss = -750, gain = 10), according to the expected value. The primary outcome of the CCT is the average number of cards chosen, which can be interpreted as a general proxy of risk-taking behavior, with a higher number of cards corresponding to greater risk-proneness. Additionally, the CCT’s normative decision-making approach suggests that participants should choose the number of cards to be turned over in accordance with their belief that the subjective value of that number of cards is maximal, in which an optimal strategy takes into account gain, loss, and risk. Therefore, the CCT also enables assessment of how much the information (i.e., gain, loss and risk) weighs on risk behaviors (i.e., number of cards) at an individual level. ^11^

### ToM: Reading the Mind in the Eyes Test (RMET)

We used the RMET to assess the ability to read others’ intentions/feelings/thoughts through affective environmental cues such as facial expressions, specifically in the region of the eyes. ^32^ In this computerized task, 36 black-and-white pictures of the same region of the face (midway along the nose to just above the eyebrows) are shown to the participants. Each image is surrounded by four words regarding mental states and participants are requested to choose the word that correctly depicts the mental state expressed in the picture. To avoid misunderstandings, a glossary is given to the participants that comprises a list of all of the words the test contains with an example of the use of each word in a phrase. ^12^ The main outcome of the RMET is the total score, which is the sum of correct answers. Nevertheless, it is also possible to investigate different subscales, splitting the items into positive, negative, and neutral emotions. ^33^

### Statistical analysis

Frequency data were analyzed with Pearson’s chi-square tests and quantitative data with Student’s *t* tests and Wilcoxon signed-rank tests, when data were non-normally distributed, Wilcoxon rank sum tests (i.e., Shapiro-Wilk W < 0.001, and skew and kurtosis divided by 2 standard errors < 2). Concerning decision-making, after performing Student’s *t* test to analyze potential group differences in overall risk-attitude independently of the decision scenario, we then extracted the risk-seeking behavior separately for the most favorable and the least favorable decision scenarios in order to assess risk-taking in a more fine-grained fashion. Differences between groups for both extreme decision scenarios and their interactions were analyzed using a linear mixed effect model (LMM), in which a two-level variable “group” (ACL and CG) and the two-level variable “decision scenario” (most favorable and the least favorable) were included as fixed effects and a random intercept was modeled for each participant. The decision scenario was established by using the expected value for each of the eight combinations of the magnitude of gain, the magnitude of loss, and probability of drawing a loss card. ^11^ Finally, to investigate how each participant weighted gains, losses, and risk information when making decisions, an LMM was performed for each individual separately including a random intercept for the three blocks and the 24 rounds in the model. Coefficients to denote how the participant weighted the gain, loss, and risk were extracted by including each one of these two-level factors as fixed-effects. This strategy has been used before and proved to be useful for differentiating people with cocaine use disorders from healthy controls. ^11^ Even though severe cognitive deficit (IQ < 65) and acute intoxication or withdrawal symptoms were used as exclusion criteria, all main analyses were performed with and without IQ, cannabis use, and CRAFFT criteria B scores as covariates. Years of school education was not included as a covariate in the models due to multicollinearity. The relationship between deliberative decision-making under risk and ToM was analyzed using Pearson’s r correlation coefficient. All statistical analyses were performed using R open-source statistical software.

## Results

### Demographic characteristics

Between-group comparisons of sociodemographic and substance use data are presented in Table 1 . Despite our efforts to keep the matching procedure, the groups differed concerning years of school education and IQ, showing that the ACL group had lower IQ and fewer years of school education. Nevertheless, as mentioned before, the analyses were performed with and without IQ, cannabis use, and CRAFFT Criteria B score as covariates, while years of school education was not included as a covariate in the models due to multicollinearity.

**Table 1:** Demographic characteristics, substance consumption, and maltreatment

	CG (n = 33)	ACL (n = 29)	Test statistics	p-value	Effect size
Demographics					
Age	16.94 (0.83)	16.76 (0.74)	*t* = 0.90	0.367	r = 0.116
Years of school education	9.76 (1.8)	7.07 (1.39)	W = 835.5	**0.000**	-
IQ	82.88 (10.49)	77.9 (7.81)	*t* = 2.13	**0.003**	r = 0.269
BMI	21.8 (3.6)	22.16 (2.58)	*t* = 0.42	0.670	r = 0.061
MMSE					
Registration	3.06 (0.35)	3 (0.00)	W = 493.5	0.348	-
Recall	2.94 (1.03)	2.76 (0.58)	W = 489.5	0.821	-
Language	7.73 (1.23)	7.9 (0.31)	W = 483.5	0.901	-
Calculation	4.73 (0.52)	4.97 (0.19)	W = 378.5	**0.020**	-
Total score	28.45 (1.28)	27.9 (1.57)	W = 581.5	0.132	-
CRAFFT					
Alcohol use (% yes)	78.00	68.00	χ ^2^ = 0.34	0.554	V = 0.112
Cannabis use (% yes)	48.00	93.00	χ ^2^ = 12.43	**0.000**	V = 0.482
Other drug use (% yes)	30.00	48.00	χ ^2^ = 1.41	0.234	V = 0.184
Criteria A score	1.38 (1.07)	2.05 (0.89)	W = 273.5	**0.014**	-
Criteria B score	2.05 (1.6)	3.2 (1.52)	W = 265.5	**0.012**	-
Criteria B (% yes)	63.00	93.00	χ ^2^ = 6.07	**0.013**	V = 0.351
CRAFFT total score	4.06 (2.32)	5.28 (2.00)	W = 343	0.052	-
JVQ-R2					
Conventional crime	5.36 (2.03)	5.34 (1.91)	W = 492.5	0.839	-
Child maltreatment	3.18 (0.92)	3.07 (1.00)	W = 505	0.689	-
Peer/sibling victimization	4.06 (1.34)	4.59 (1.12)	W = 366.5	0.102	-
Indirect victimization	3.79 (1.83)	3.24 (1.72)	W = 557	0.259	-
Sexual victimization	6.33 (0.85)	5.86 (1.03)	W = 590	0.086	-
ICU					
Callousness	6.06 (2.41)	5.77 (3.98)	*t* = 0.27	0.783	r = 0.046
Uncaring	6.72 (3.82)	6.27 (3.83)	*t* = 0.36	0.713	r = 0.061
Unemotional	7.67 (3.4)	6.95 (3.93)	*t* = 0.61	0.542	r = 0.099
Total score	19.17 (5.64)	17.64 (7.85)	*t* = 0.715	0.478	r = 0.116

ACL = adolescents in conflict with the law; BMI = body mass index; CG = control group; ICU = Inventory of Callous-Unemotional Traits; JVQ-R2 = Juvenile Victimization Questionnaire second revision; MMSE = Mini Mental State Examination; IQ = Estimated Intelligence Quotient ± 20. Bold type denotes statistical significance.

CRAFFT Criteria A assesses whether the participant used the substance. CRAFFT Criteria B refers to the magnitude of problems associated with substance use.

We found that ACL performed better than controls in the MMSE calculation score. Taken together with the overall IQ, this observed difference in the MMSE calculation score (based on the median) could suggest that ACL may perform better in tasks that require more fluid intelligence than crystallized intelligence. This would indicate that, despite their educational disadvantage, ACL were still able to perform basic math slightly better than controls. Nevertheless, this hypothesis extrapolates the data and does not seem to be in accordance with the literature, which has found that adopted juvenile delinquents scored lower in the arithmetic subscale of the Wechsler Intelligence Scale for Children compared to the population mean. ^34^ It should be noted, however, that both controls and ACL still performed within the expected average in our study. In general, ACL reported more cannabis use and more problems related to substance use when compared to controls, with most of the ACL group being at risk for substance use disorder. No differences were found for juvenile victimization or antisocial personality traits.

### ToM

**Figure 1:**
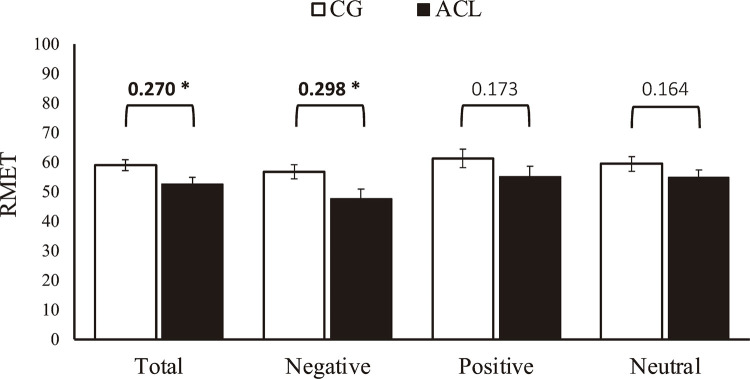
Effect sizes were calculated using Pearson’s correlation coefficients (r < 0.10 small effect size; r < 0.30 medium effect size; r < 0.50 large effect size. ACL = adolescents in conflict with the law; CG = control group; RMET = Reading the Mind in the Eyes Test. * p < 0.05.

In general, our RMET data ( Figure 1 ) suggest that the ACL differ from CG concerning ToM, as depicted by the group differences in the total score ( *t*_58.09_ = 2.14, p = 0.036). However, this finding is mainly explained by group differences in the ability to correctly infer negative emotional states ( *t*_55.36_ = 2.32, p = 0.023), since no significant group differences were found in the positive ( *t*_58.64_ = 1.35, p = 0.181) or neutral ( *t*_59.57_ = 1.28, p = 0.204) subscales. When IQ, cannabis use, and CRAFFT Criteria B were included in the model, neither group differences in total score nor in the negative subscale score remained. However, an effect was found for IQ in the total score ( *t*_57_ = 2.08, p = 0.041, r = 0.082), while an effect for cannabis use was found in the negative subscale ( *t*_57_ = -2.07, p = 0.042, r = 0.264).

### Deliberative decision-making under risk.

**Figure 2:**
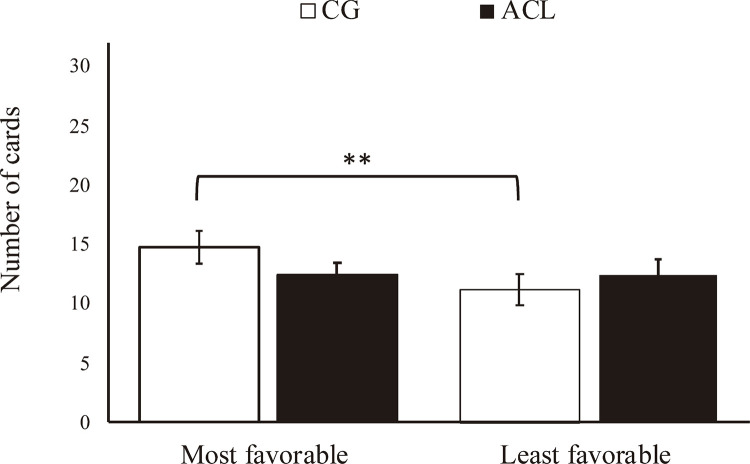
ACL = adolescents in conflict with the law; CG = control group. ** p < 0.01. Finding still significant with years of school education, Mini-Mental State Examination (MMSE) total score, and CRAFFT Criteria A score included in the model (p < 0.05).

No significant group differences were found regarding the overall number of cards chosen, suggesting that groups did not differ concerning general risk-taking behavior ( *t*_53.73_ = -0.08, p = 0.935, r = 0.011). Nonetheless, a more detailed inspection applying LMM to the most favorable and the least favorable decision scenarios revealed an effect for the decision scenario, suggesting that, in general, participants chose less cards in the least favorable scenario when compared to the most favorable scenario (β = -0.51, *t*_60_ = -3.14, p = 0.002, r = 0.376). An interaction between the ACL group and decision scenario was found (β = 0.50, *t*_60_ = 2.08, p = 0.040, r = 0.260), which prompted us to perform multiple-comparison tests. As shown in Figure 2 , the post-hoc Bonferroni pairwise comparisons revealed that only the CG members were able to adapt behavior from the most favorable decision scenarios to the least favorable one (β = 3.60, *t*_60_ = 3.14, p = 0.002, r = 0.376), according to the available information and, consequently, the expected value. These results remained significant even when including IQ, cannabis use, and CRAFFT Criteria B score. Taking all the LMM findings together, they suggest that the CG adolescents were able to deliberatively modulate behavior based on the environmental information, taking more risks when the situation was advantageous and taking fewer risks when the situation was disadvantageous.

**Figure 3:**
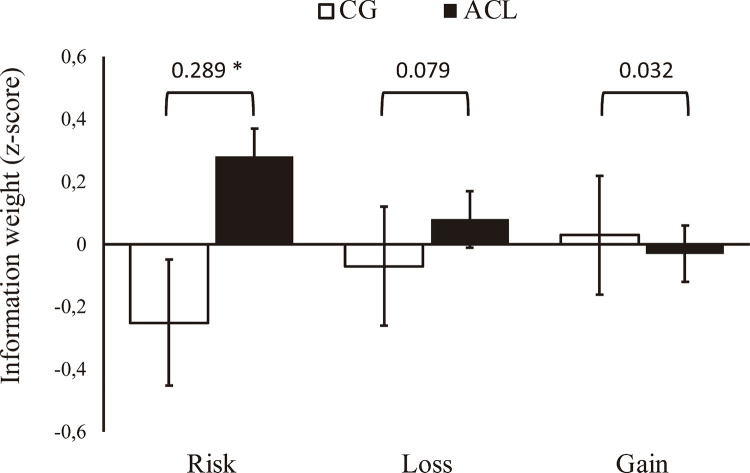
No significant effect for risk information was found when years of school education, Mini-Mental State Examination (MMSE) total score, and CRAFFT Criteria A score were included in the model. * p < 0.05.

Use of risk, loss, and gain information was extracted for all participants individually. Subsequently, group averages were compared and a group effect was found for risk information only ( Figure 3 ). Due to the small sample size and lack of power, no significant difference between groups was found concerning use of loss and gain information, as shown in Figure 3 . Remarkably, the groups showed distinct patterns of use of information. While the risk and loss information had a negative effect on the number of cards chosen by the CG adolescents, as would be expected, we observed that the same information tends to increase the number of cards chosen by members of the ACL group. Moreover, as shown by the effect sizes, the most used information was risk, followed by loss and gain, successively. These results remained significant even when including IQ, cannabis use, and CRAFFT Criteria B score.

### Decision-making and ToM

Finally, concerning the relationship between deliberative decision-making under risk and affective inference of emotional states through ToM, no correlation was found between the main CCT and RMET outcomes.

## Discussion

Here we examined the differences between ACL and a CG regarding deliberative decision-making under risk, using the CCT, and ToM, assessed using the RMET. The key findings are that ACL differed from controls concerning their capacity to properly infer emotional states in others and to make decisions under risk scenarios. Specifically, we found that ACL committed more errors when inferring negative emotional states, but no difference was found in the error rate concerning neutral and positive emotional states. With regards to deliberative decision-making, our results suggest that the ACL group did not vary their behavior based on the available information and that the risk information has an opposite effect on the number of cards (risk-taking behavior) they choose when compared to the CG. Our main findings were not better explained by group differences with regards to general cognitive ability, assessed using years of school education and IQ, or substance use, assessed with the CRAFFT scale. Finally, as expected, no correlation was found between the RMET and CCT main outcomes, suggesting that performances in the tasks are independent of each other and, therefore, may depict two distinct cognitive processes. To our knowledge, this is the first study to investigate the performance in both social cognition and decision-making under risk of ACL currently subject to provisional deprivation of liberty.

In general, our findings could be discussed considering dual-processing models that have proposed that the adolescent risk-taking profile results, at least partially, from a developmental imbalance between two complementary neural systems. The affective system relies on midbrain dopaminergic centers, such as the ventral tegmentum area and both subcortical structures (amygdala and ventral striatum) and cortical structures (medial and orbital regions of the frontal cortex and the insular cortex). The cognitive-control system relies on the dorsal and ventral portions of the lateral prefrontal cortex and posterior parietal cortex. ^35^ Therefore, while the affective system’s responsiveness develops rapidly at puberty, the deliberative cognitive-control system matures more gradually over the course of adolescence and young adulthood. Consequently, when faced with making deliberative decisions, such as the ones elicited by the CCT, adolescents may struggle to properly perceive uncertainty about outcomes and evaluate possible benefits or costs for the physical, economic, or psycho-social well-being of themselves or others. ^30^ Moreover, when facing affective contexts, such as those that require social cognitive abilities, as in the RMET, the active affective neural system may be impaired, overloaded, or even blunted. ^36 , 37^ Together, our findings could suggest that ACL may have even later maturation or neurodevelopmental abnormalities in these systems. Nevertheless, such conclusions should be treated with extreme caution considering that we have only analyzed behavioral data here.

Specifically concerning our ToM findings, it has been demonstrated that impaired ToM is a predictor of behavioral problems in children aged between 7 and 11 years. ^38^ Similarly, it was also found to be a predictor of a lack of appreciation of social rules and norms, especially when associated with psychopathologies. ^39 , 40^ Nevertheless, whether ToM can explain an individual’s propensity to delinquent behavior is under debate. On one hand, Majorek et al. ^39^ argue that ToM skills are neither necessary nor sufficient to explain an individual’s propensity to delinquent behavior and a review concluded that a relationship between empathy and adolescent offending could not be confirmed. ^41^ On the other hand, a previous study employed a ToM-based intervention in a single-subject design with incarcerated youth offenders that showed improvement in social problem-solving. ^42^ It may be the case that is not a pivotal piece in the emergence of delinquent behavior, but is a trigger during development. There is no data in the literature supporting clear differences between people with a history of legally relevant aggressive behavior and psychopathic traits regarding ToM, ^43 - 45^ but there is evidence supporting the view that impairments in this regard reduce during development. ^46^ Interestingly, a study conducted by Jusyte and Schönenberg ^47^ revealed a specific deficit in the categorization of ambiguous negative facial expressions in a group of violent offenders, while no differences were observed concerning their perceptual sensitivity to neutral/emotional stimuli. Their findings suggest that violent offenders may show a deficit tied to perception of negative emotional states, but not necessarily related to social information processing.

Regarding decision-making, our main finding was not about general risk-taking behavior but revealed a clear difficulty of the ACL group to adapt the expected behavior based on the available information, mainly in response to information related to risk, but not to gain or loss. In other words, what we observed was a poor ability to distinguish subtle differences between favorable and unfavorable/negative decision-making scenarios associated with risky options. Our data corroborate a previous review that suggested that impairments in risky decision-making tasks may indeed be more profound than those in other neuropsychological tasks for offenders, which is an important predictor for cognitive behavioral interventions. ^48^ Nonetheless, altogether, our data also suggest that rather than exhibiting widespread deficits in ToM and deliberative decision-making, ACL tend to show an insufficient ability to discriminate ambiguous negative stimuli. This is relevant considering that experiencing negative outcomes might increase risk perception while failing to experience negative outcomes might have the opposite effect. ^49^ In this sense, one might argue that poor negative stimuli processing could impair learning rate during the transition between childhood to adolescence, which could be associated with some immaturity in dealing with social, emotional, and risky situations, ultimately increasing exploration and risk-taking behaviors. Based on this rationale and in the context of our experiment – which used the no-feedback version of the CCT in which participants cannot monitor their performance during the experiment ^1^ – it is possible to speculate that if participants had received feedback on whether they answered correctly in the RMET, or whether they won or lost in each CCT trial, they could have improved their gist representation of risk and, consequently, could have performed better. However, in a previous study we have already shown that even when exposed to feedback in the same task, female adolescents tend not to adapt their behavior accordingly. ^30^ Furthermore, the present study corroborates a previous finding that poor performance in the Iowa Gambling Task (IGT), a task that requires implicit learning and a gist representation of risk, can predict recidivist offenders and total lifetime incarceration. ^50^ The authors explained that recidivist offenders showed a distinct pattern of IGT performance characterized by a failure to learn from feedback or to modify their preferences to more advantageous decks of cards.

Additionally, it is worth mentioning that our group effects regarding ToM did not remain significant when cannabis use and IQ were included in the model. Intriguingly, it has already been demonstrated that cannabis use can affect processing of social information, which might be a possible risk factor for psychosis. ^51^ Moreover, neurocognition tests, mainly executive functioning that is strongly related to IQ, were associated with ToM and social cognition. ^52^ Hence, although it is very risky to conclude the following explanation by extrapolating our data, it can be hypothesized that substance use and a reduced IQ may exacerbate general ToM inabilities in our sample, which, combined with an impairment in properly pondering risk information and adapting risk-taking behaviors accordingly, could increase the likelihood of becoming involved in conflicts with the law. Although this hypothesis is beyond what we can prove here, it suggests that delinquent behaviors and conflict with the law are indeed multifactorial and depend not only on sociodemographic vulnerabilities but also on individual factors such as substance use and general cognition. Future studies could go further into this hypothesis by implementing an in-depth and precise assessment of substance consumption and cognitive performance, including an entire decision-making neuropsychological test battery.

Our paper has some limitations to be considered. First, our sample is small. However, we recruited from a very specific population of adolescents who were under provisional deprivation of liberty in social rehabilitation centers. To gain access to this sample, the project went through two different ethics committees and researchers had to attend to a strict set of ethical concerns. Therefore, a second limitation is related to the resulting need for a short protocol, which did not allow us to go further and investigate psychiatric symptoms and disorders. Nevertheless, we were able to assess juvenile victimization and callous and unemotional personality traits, but, interestingly, no differences in these regards were found between groups. This finding suggests that, at least in our sample, delinquent behavior seems to be more related to social and non-social categorization of negative stimulus than to an intrinsic personality profile. Certainly, considering that some studies have already shown that anxiety symptoms and stress may affect value-based decision-making, ^53 - 55^ future studies might fill this gap. Finally, it is important to highlight that most of the participants excluded were removed from the sample because of severe cognitive deficit - approximately 35% from the ACL group and around 10% from the CG. These proportions cannot be seen as mere sample losses, but they may also suggest that the explanation of why adolescent conflicts with the law go far beyond a specific deficit in evaluating negative stimuli. Nonetheless, additional studies are needed in this regard.

In conclusion, although we should acknowledge the multitude of environmental factors that can influence criminal choice, here we found that, compared to CG adolescents, ACL presented poorer processing of negative emotional states and reduced behavioral adaptation capacity based on available risk information. Moreover, it is well-accepted that cognitive skills, even those such as decision-making, are complex behaviors that develop and depend on environmental experiences throughout childhood and adolescence, and that can still be changed through lifelong learning alongside the biological predisposition. Therefore, our findings suggest that ACL may present an insufficient ability to discriminate negative stimuli, rather than widespread deficits in ToM and deliberative decision-making. This has important implications for development of interventions for these adolescents, suggesting that they tend to learn little from negative outcomes and have reduced capacity to process negative emotions. Consequently, the current findings suggest that a punitive system based on negative stimuli, for instance, may not be effective in terms of educational measures, since there is evidence that punishment does not lead to enhancement of learning in human subjects. ^56^ Additionally, it is possible that educational strategies that sensitize teenagers to think about the consequences of their actions, such as sports, or even board games like chess, focus groups, and empathy training, could add to the efficacy of these youths’ rehabilitation. Moreover, one may also think of preventive strategies, for instance, since improving ToM skills in people in situations of social vulnerability could be beneficial for their development. In any case, further studies could be performed that combine both basic and applied cognitive science.
